# Microperimetric evaluation for different methods of epiretinal membrane surgery

**DOI:** 10.1186/s12886-023-03056-3

**Published:** 2023-06-29

**Authors:** Seung Wan Nam, Zeeyoon Byun, Don-Il Ham, Mingui Kong

**Affiliations:** 1 Department of Ophthalmology, HanGil Eye Hospital, Incheon, Korea; 2 Department of Ophthalmology, College of Medicine, Catholic Kwandong University, Incheon, Korea; 3 Department of Ophthalmology, Samsung Medical Center, Sungkyunkwan University School of Medicine, Seoul, Korea; 4 Department of Ophthalmology, Kangbuk Samsung Hospital, Sungkyunkwan University School of Medicine, 29, Saemunan-ro, Jongno-gu, Seoul, Korea

**Keywords:** Epiretinal membrane, Internal limiting membrane removal, Indocyanine green, Microperimetry

## Abstract

**Purpose:**

To investigate the anatomic and functional outcomes using microperimetry for the surgical methods for idiopathic epiretinal membranes (ERM).

**Methods:**

This retrospective study included 41 eyes from 41 patients. All patients underwent combined epiretinal membrane and cataract surgery. Best-corrected visual acuity (BCVA), optical coherence tomography, and microperimetry were performed before and 6 months and 1 year after surgery. The patients were divided into 3 groups; “ERM removal only without indocyanine green (ICG) staining”, “ERM and internal limiting membrane (ILM) removal without ICG staining”, and “ERM and ILM removal with ICG staining”.

**Results:**

Preoperatively, the ages, BCVAs, central macular thickness (CMT), and mean retinal sensitivities of central 6° (MRSs) of the groups were not significantly different (*p* > 0.05). Postoperatively, the MRSs of the “ERM removal only without ICG staining” and “ERM and ILM removal without ICG staining” groups were not significantly different (*p* > 0.05). The MRSs of the “ERM and ILM removal without ICG staining” and “ERM and ILM removal with ICG staining” groups were not significantly different (*p* > 0.05). However, the MRSs of the “ERM and ILM removal with ICG staining” group significantly reduced than “ERM removal only without ICG staining” group (*p* < 0.05).

**Conclusion:**

This retrospective study found reduced retinal sensitivity in ERM and ILM removal with ICG staining group compared to ERM removal only without ICG staining. Further studies with larger sample sizes are required.

## Introduction

Idiopathic epiretinal membrane (ERM) is a relatively common disorder in older adults [1]. Idiopathic ERM is associated with macular dysfunction related to an impairment of the inner retina [2]. Most patients with idiopathic ERM are asymptomatic, but they can complain of metamorphopsia and decreased visual acuity [3]. Pars plana vitrectomy (PPV) is the gold standard for treating symptomatic idiopathic ERM, [4] and several methods have been suggested [2]. Among the suggested PPV methods, peeling of the internal limiting membrane (ILM) remains controversial. ILM peeling may have a lower recurrence rate of ERM and better visual acuity after surgery [5, 6]. However, ILM peeling can cause macular holes due to Müller cell damage, [7] nerve fiber layer damage, retinal hemorrhages, retinal edema, electrophysiological changes, and visual field changes [8].

Intraocular dyes, including indocyanine green (ICG), for PPV help surgeons to visualize ILM during ILM peeling [2]. However, ICG dyes may have adverse effects on functional outcomes, including vision and visual field [9]. The suggested mechanisms of ICG toxicity include injury to the ganglion and neuroretinal cells, RPE cells, and superficial retinal vessels [10]. These may be mediated by apoptosis, gene expression, osmolarity effect, phototoxicity, and direct injury [10]. Therefore, the use of the ICG dye during PPV for idiopathic ERM is still controversial.

Microperimetry is a non-invasive and effective method for the detection of functional changes [11]. It can assess visual field defects topographically and is a useful tool, in that the shape of the macula is not always associated with the function of the macula [12]. Microperimetric assessment of epiretinal membrane surgery has been reported [3]. However, microperimetric differences between surgical methods for ERM have not yet been reported.

The COMPASS fundus perimeter (CMP) has the advantage of assessing patients with overimposed fundus and retinal sensitivity images, allowing simultaneous assessment of retinal function and structure [13]. Therefore, CMP can be used to assess patients who underwent ERM surgery with precision to correlate the ERM/ILM peeling site and retinal sensitivity.

This study aimed to investigate the effects of ILM peeling and the toxicity of ICG dyes on microperimetry during PPV for idiopathic ERM.

## Methods

This study was approved by the Institutional Review Board (IRB) of HanGil Eye Hospital and adhered to the tenets of the Declaration of Helsinki. Given the retrospective design of this study and the use of anonymized data, the requirement for informed consent was waived by the IRB of the HanGil Eye Hospital in Korea.

This retrospective study included patients who underwent PPV for idiopathic ERM between June 2020 and June 2021 at the HanGil Eye Hospital. Only phakic patients were enrolled in this study. The exclusion criteria were secondary ERM, pseudophakia, uveitis, severe diabetic retinopathy, severe hypertensive retinopathy, retinal detachment, glaucoma, refractive error exceeding ± 6 diopters, history of retinal laser photocoagulation, severe ocular media opacity, and insufficient ocular examinations. Patients were classified into three groups based on surgical methods; “ERM removal only without ICG staining,” “ERM and ILM removal without ICG staining,” and “ERM and ILM removal with ICG staining”.

Preoperatively, all patients underwent a complete ophthalmologic examination, including slit-lamp examination and measurement of best-corrected visual acuity (BCVA), color fundus photography (CFP; TrueColor Confocal slit scanner, Centervue Spa, iCare Finland Oy, Vantaa, Finland), spectral-domain optical coherence tomography (SD-OCT; Spectralis HRA + OCT, Heidelberg Engineering, Heidelberg, Germany), and microperimeter (Compass fundus perimeter, CMP; Centervue Spa, a company of iCare Finland Oy; Vantaa, Finland). Patient information, including age, sex, and microperimetric parameters, was obtained for each eye.

Postoperatively, all patients were followed up after 6 months and 1 year. BCVA, SD-OCT, and CMP assessments were repeated to evaluate the anatomic and functional outcomes.

### Surgical technique

All patients underwent combined epiretinal membrane and cataract surgery to minimize the effect of cataract progression on retinal sensitivity after PPV. We performed standard phacoemulsification for the intraocular lens surgery followed by standard 3 port PPV (25 gauge). All patients underwent in-the-bag implantation of ARTIS® PL E (Cristalens Industrie, Lannion, France). Balanced salt solution (BSS; Alcon Laboratories, Fort Worth, TX, USA) was used as the irrigation solution. Induction of posterior vitreous detachment was performed, if it had not been done. During the PPV for the “ERM removal only without ICG staining” group, the ERM was engaged with intraocular forceps to create a flap and then peeled. In the “ERM and ILM removal without ICG staining” group, ERM was peeled the same way, and triamcinolone (TA) was used to facilitate the removal of ILM. Subsequently, the ILM was peeled with the intraocular forceps, which was initiated by creating a flap. In the “ERM and ILM removal groups with ICG staining” group, the ERM was peeled the same way. Irrigation was terminated, and less than 0.5 ml of ICG (Pulsion Medical Systems AG, Munich, Germany) at a 0.05% concentration dissolved in dextrose 5% was injected into the BSS-filled globe just above the posterior pole. After 30 s, irrigation was restarted and irrigation with ICG was performed until the ICG dye was invisible. ICG staining was performed only once. The green-stained ILM was engaged using intraocular forceps and peeled in the same manner. No tamponade agents were used during surgery. After initiation of the flap, ILM and ERM were grasped with intraocular forceps. The ILM and ERM were peeled from an area within 2 disc diameters from the fovea. Complete removal of the ERM was attempted in all patients in all groups. All the surgeries were performed by the same skilled surgeon (M.K.).

### Imaging protocols

The CFP was performed with an angular range of approximately 60° horizontally and 55° vertically. The automatic real-time (ART) mode was activated using an eye-tracker system during SD-OCT. The protocol of SD-OCT consisted of two B-scans centered on the fovea (horizontal and vertical, 12.0 mm, ART 100) and raster scans (30° x 20°, 6.0 mm, centered at the center of the fovea, 25 horizontal B-scans, ART 9). Central macular thickness (CMT) was measured at the circular area 1 mm centered to the fovea, acquired from 3D scan protocol, given by the automated software of SD-OCT.

### Microperimetry

**Fig. 1:**
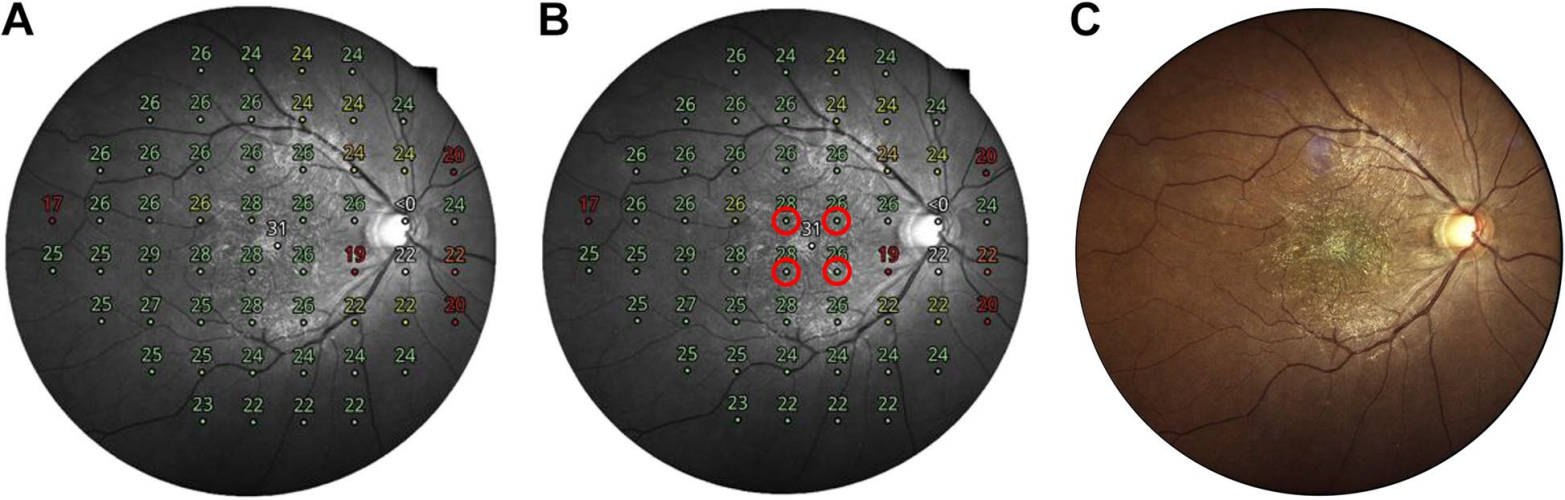
Measurement of retinal sensitivity using microperimetry. Microperimetry was performed using a compass fundus microperimeter with a standard 24 − 2 grid. Mean retinal sensitivity (MRS), defined as the arithmetic average of the retinal sensitivities of the nearest four points from the fovea in each test, was manually calculated. **A** Composited images of topographical information on retinal sensitivity and red free fundus photographs were obtained. **B** Retinal sensitivities of the nearest four points from the fovea were recorded and MRS was calculated. **C** A color fundus photograph was used to confirm that the ERM/ILM peeled area matched the nearest four points from the fovea

Microperimetry was performed using CMP. All patients underwent mesopic tests. Prior to testing, pupillary dilation was performed using 1.0% tropicamide. The room light was switched off immediately before each examination. The standard 24 − 2 grid was used in this study. However, we only analyzed the retinal sensitivity of the nearest four points from the fovea, which covers the central 6° to correlate with the ERM/ILM peeled area. These points were classified into superonasal (SN), inferonasal (IN), superotemporal (ST), and inferotemporal (IT) areas, according to their location. The mean retinal sensitivity (MRS), defined as the arithmetic average of the retinal sensitivities of the nearest four points from the fovea in each test, was manually calculated. The testing strategy was Zippy estimation by sequential testing (ZEST) [14]. ZEST is a perimetric algorithm with reasonable error and test time [14, 15]. Microperimetry tests were considered reliable if the false-positive rate was less than 18% [14]. Active compensation for fixation loss was provided by automated, tracking of eye movements by infrared scanning of the retina. The superimposed fundus image automatically generated by CMP was used to confirm that the ERM/ILM peeled area was matched with the nearest four points from the fovea. (Fig. 1). Superimposed fundus images are composite of topographical information on retinal sensitivity and red free fundus photographs.

### Statistical analysis

Quantitative variables are presented as the mean ± standard deviation. Frequencies were compared between the groups using the chi-square test. Analyses of continuous variables between groups were performed using independent t-tests, paired t-tests, and one-way analysis of variance (ANOVA) tests. An independent t-test was used for comparisons between two groups, and one-way ANOVA was used for comparisons among three groups. A paired t-test was used to compare postoperative and preoperative values.

Statistical significance was defined as a *p*-value < 0.05. All statistical analyses were performed using the SPSS software (version 20.0; SPSS, Chicago, IL, USA).

## Results

A total of 41 eyes of 41 patients were included in the study. During the baseline examination, the mean age was 67.3 ± 4.8 years, and 17/41 (41.5%) patients were male. The refractive error was − 0.28 ± 1.59 diopters, BCVA was 0.42 ± 0.20 logMAR (0.42 ± 0.18 Decimal equivalent; 20/47.62 ± 20/111.11 Snellen equivalent), and intraocular pressure was 15.02 ± 2.40 mmHg. All patients were phakic (100.0%). CMT was 443.8 ± 77.0 μm.

### Analyses of baseline characteristics according to the surgical methods

The patients were divided into three 3 groups according to the surgical methods. Twelve eyes were enrolled in the “ERM removal only without ICG staining” group, 16 eyes were enrolled in the “ERM and ILM removal without ICG staining” group, and 13 eyes were enrolled in the “ERM and ILM removal with ICG staining” group.

The ages at the first exam were 66.8 ± 4.1 years, 67.1 ± 5.9 years, and 67.8 ± 4.1 years for the “ERM removal only without ICG staining”, “ERM/ILM removal without ICG staining”, and “ERM/ILM removal with ICG staining” groups, respectively, and these were not significantly different according to the ANOVA test (*p* = 0.865) and independent t-tests (*p* > 0.05).

The BCVAs at the first exam were 0.43 ± 0.25 logMAR (0.43 ± 0.20 Decimal equivalent; 20/46.51 ± 20/100 Snellen equivalent), 0.40 ± 0.19 logMAR (0.43 ± 0.20 Decimal equivalent; 20/46.51 ± 20/100 Snellen equivalent), and 0.44 ± 0.18 logMAR (0.40 ± 0.15 Decimal equivalent; 20/50 ± 20/133.33 Snellen equivalent) for the “ERM removal only without ICG staining,” “ERM/ILM removal without ICG staining,” and “ERM/ILM removal with ICG staining” groups, respectively, and these were not significantly different according to the ANOVA test (*p* = 0.890) and independent t-tests (*p* > 0.05).

The MRSs at the first exam were 26.60 ± 1.11 dB, 25.89 ± 1.26 dB, and 26.00 ± 0.91 dB for the “ERM removal only without ICG staining,” “ERM/ILM removal without ICG staining,” and “ERM/ILM removal with ICG staining” groups, respectively, and these were not significantly different according to the ANOVA test (*p* = 0.228) and independent t-test (*p* > 0.05).

The CMT at the first exam were 442.1 ± 64.2 μm, 444.4 ± 74.3 μm, and 444.6 ± 95.4 μm for the “ERM removal only without ICG staining,” “ERM/ILM removal without ICG staining,” and “ERM/ILM removal with ICG staining” groups, respectively, and these were not significantly different according to the ANOVA test (*p* = 0.996) and independent t-test (*p* > 0.05).

The proportions of males, refractive error, and intraocular pressure were not significantly different in the groups according to the ANOVA and independent t-tests (*p* > 0.05). The baseline characteristics of the patients in the three groups are summarized in Table 1.

**Table 1:** Baseline characteristics of epiretinal membrane surgery according to the surgical methods

	ERM only s ICG (*n* = 12)	ERM/ILM s ICG (*n* = 16)	ERM/ILM c ICG (*n* = 13)	p_12_	p_23_	p_13_	p_*_
Age at first exam (years)	66.8 ± 4.1	67.1 ± 5.9	67.8 ± 4.1	0.879	0.702	0.545	0.865
Male (%)	6/12 (50.0%)	8/16 (50.0%)	3/13 (23.1%)	1.000	0.137	0.161	0.266
Refractive error (D)	-0.79 ± 1.61	-0.01 ± 1.71	-0.15 ± 1.41	0.226	0.803	0.305	0.416
BCVA (logMAR)	0.43 ± 0.25	0.40 ± 0.19	0.44 ± 0.18	0.782	0.601	0.894	0.890
Intraocular pressure (mmHg)	14.83 ± 1.99	15.31 ± 2.68	14.85 ± 2.54	0.592	0.636	0.989	0.835
Lens status (phakia)	12/12 (100.0%)	16/16 (100.0%)	13/13 (100.0%)	N/A	N/A	N/A	N/A
CMT (µm)	442.1 ± 64.2	444.4 ± 74.3	444.6 ± 95.4	0.931	0.994	0.938	0.996

Data are total no. (%) or mean ± standard deviation, unless otherwise indicated

*ERM* epiretinal membrane, *ILM* internal limiting membrane, *ICG* indocyanine green dye, *D* diopters, *BCVA* best corrected visual acuity, *ERM only s ICG* ERM removal only without ICG staining group, *ERM/ILM s ICG* ERM/ILM removal without ICG staining group, *ERM/ILM c ICG* ERM/ILM removal with ICG staining group, *logMAR* logarithm of the minimum angle of resolution, *N/A* not applicable, *CMT* central macular thickness

p12 = *p*-value of comparison between ERM only s ICG group and ERM/ILM s ICG group; p23 = *p*-value of comparison between ERM/ILM s ICG group and ERM/ILM c ICG group; p13 = *p*-value of comparison between ERM only s ICG group and ERM/ILM c ICG group; p* = *p*-value of comparison between all groups

### Surgical outcomes of combined epiretinal membrane and cataract surgery

Overall, patients showed improvement in CMT (443.8 ± 77.0 μm vs. 378.0 ± 50.5 μm, *p* = 0.000), BCVA (0.42 ± 0.20 logMAR (0.42 ± 0.18 Decimal equivalent; 20/47.62 ± 20/111.11 Snellen equivalent) vs. 0.15 ± 1.10 logMAR (0.73 ± 0.16 Decimal equivalent; 20/27.40 ± 20/125 Snellen equivalent), *p* = 0.000), and MRS (26.13 ± 1.13 dB vs. 26.85 ± 1.53 dB, *p* = 0.007) 1 year after surgery. The intraocular pressures were not different between the preoperative and postoperative exams after 1 year (15.02 ± 2.40 mmHg vs. 14.73 ± 2.50 mmHg, *p* = 0.349).

**Fig. 2:**
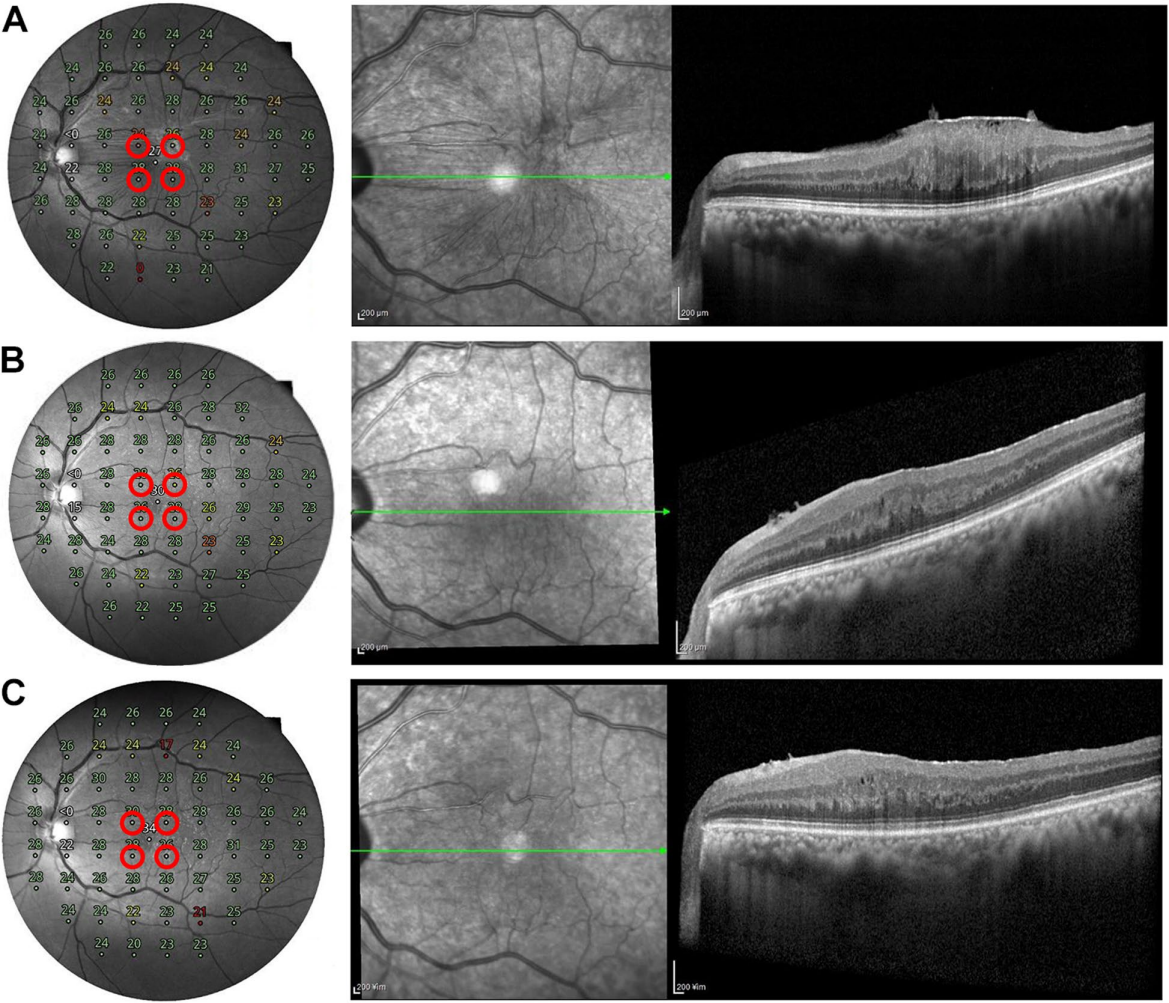
A representative case of complete removal of epiretinal membrane (ERM) after surgery. A 58-year-old man presented with an idiopathic ERM. The patient underwent combined ERM and cataract surgery. He was classified into “ERM and ILM removal without ICG staining” group. **A** Preoperative spectral-domain optical coherence tomography (SD-OCT) showing ERMs with macular edema. The mean retinal sensitivity (MRS) of the nearest 4 points from the fovea was 26.5 decibel (dB). **B **At 6 months after surgery, removal of the ERM was confirmed in the SD-OCT image. The MRS of the nearest 4 points from the fovea was 27.0 dB. **C **One year after surgery, there was no recurrence of ERM in the SD-OCT image. The MRS of the nearest 4 points from the fovea was 28.0 dB

**Fig. 3:**
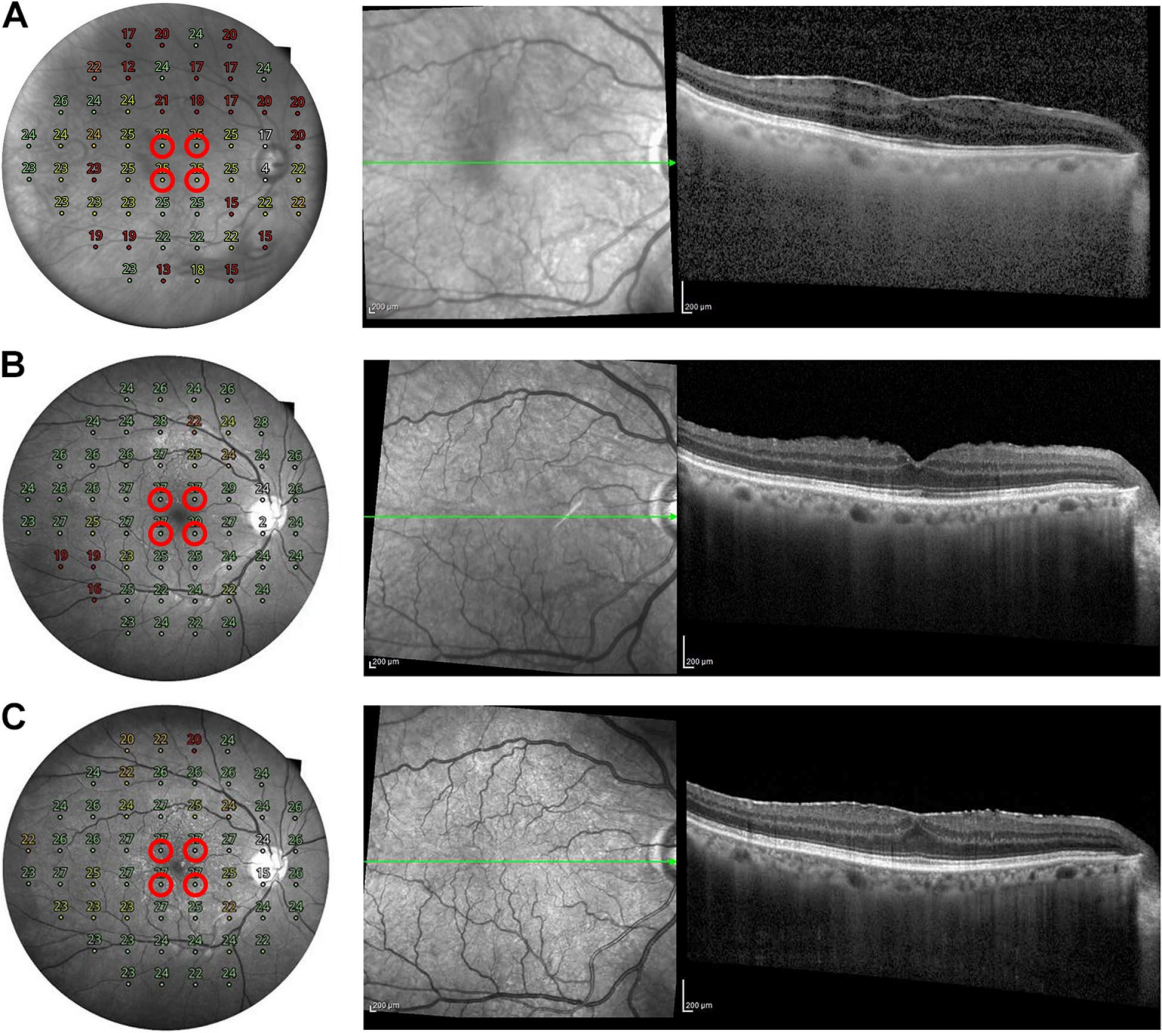
A representative case of recurred epiretinal membrane (ERM) after surgery. A 66-year-old man presented with an idiopathic ERM. The patient underwent combined ERM and cataract surgery. He was classified into “ERM removal only without ICG staining” group. **A** Preoperative spectral-domain optical coherence tomography (SD-OCT) showing ERMs with macular edema. The mean retinal sensitivity (MRS) of the nearest 4 points from the fovea was 25.0 decibel (dB). **B** At 6 months after surgery, removal of the ERM was confirmed in the SD-OCT image. The MRS of the nearest 4 points from the fovea was 27.5 dB. **C** One year after surgery, ERM recurred in the SD-OCT image. The MRS of the nearest 4 points from the fovea was 27.0 dB

Complications of PPV, including retinal breaks and detachment, glaucoma, and endophthalmitis, have not yet been reported. Any signficant posterior capsular opacition was not observed. ERM recurred in 2 patients in the “ERM removal only without ICG staining” group. The other groups did not show ERM recurrence after surgery. Representative cases are presented below (Figs. 2 and 3).

### Analyses of outcomes according to the surgical methods

At the postoperative 6-month follow-up visit, the CMT of the “ERM removal only without ICG staining”, “ERM/ILM removal without ICG staining”, and “ERM/ILM removal with ICG staining” groups were 404.5 ± 51.0 μm, 389.2 ± 58.2 μm, and 401.1 ± 47.3 μm, respectively, and these were not significantly different according to the ANOVA test (*p* = 0.719) and independent t-test (*p* > 0.05).

At the postoperative 1-year follow-up visit, the CMT of the “ERM removal only without ICG staining”, “ERM/ILM removal without ICG staining”, and “ERM/ILM removal with ICG staining” groups were 391.1 ± 42.0 μm, 373.5 ± 53.1 μm, and 371.3 ± 55.6 μm, respectively, and these were not significantly different according to the ANOVA test (*p* = 0.571) and independent t-tests (*p* > 0.05).

At the postoperative 6-month follow-up visit, the BCVAs of the “ERM removal only without ICG staining”, “ERM/ILM removal without ICG staining”, and “ERM/ILM removal with ICG staining” groups were 0.15 ± 0.07 logMAR (0.72 ± 0.11 Decimal equivalent; 20/27.78 ± 20/181.8 Snellen equivalent), 0.16 ± 0.10 logMAR (0.71 ± 0.17 Decimal equivalent; 20/28.17 ± 20/117.65 Snellen equivalent), and 0.19 ± 0.14 logMAR (0.68 ± 0.21 Decimal equivalent; 20/29.41 ± 20/95.24 Snellen equivalent), respectively, and these were not significantly different according to the ANOVA test (*p* = 0.659) and independent t-test (*p* > 0.05).

At the postoperative 1-year follow-up visit, the BCVAs of the “ERM removal only without ICG staining”, “ERM/ILM removal without ICG staining”, and “ERM/ILM removal with ICG staining” groups were 0.13 ± 0.05 logMAR (0.74 ± 0.09 Decimal equivalent; 20/27.03 ± 20/222.22 Snellen equivalent), 0.15 ± 0.09 logMAR (0.72 ± 0.16 Decimal equivalent; 20/27.78 ± 20/125 Snellen equivalent), and 0.15 ± 0.15 logMAR (0.74 ± 0.23 Decimal equivalent; 20/27.03 ± 20/86.96 Snellen equivalent), respectively, and these were not significantly different according to the ANOVA test (*p* = 0.874) and independent t-tests (*p* > 0.05).

At the postoperative 6-month follow-up visit, the MRSs of the “ERM removal only without ICG staining,” “ERM/ILM removal without ICG staining,” and “ERM/ILM removal with ICG staining” groups were 27.56 ± 1.23 (dB), 26.45 ± 1.92 (dB), and 25.73 ± 1.37 (dB), respectively. There were significant differences according to the ANOVA test results for three groups (*p* = 0.022) and independent t-test results for the “ERM removal only without ICG staining” and “ERM/ILM removal with ICG staining” groups (*p* = 0.002), but there was no difference between the other two groups according to the independent t-tests (*p* > 0.05).

At the postoperative 1-year follow-up visit, the MRSs of the “ERM removal only without ICG staining,” “ERM/ILM removal without ICG staining,” and “ERM/ILM removal with ICG staining” groups were 27.56 ± 0.72 (dB), 26.83 ± 2.08 (dB), and 26.23 ± 1.01 (dB), respectively, and these were not significantly different according to the ANOVA (*p* = 0.092) and independent t-test results between the groups (*p* > 0.05), except between the “ERM removal only without ICG staining” and “ERM/ILM removal with ICG staining” groups (*p* = 0.001).

Table 2 shows the results of the comparative analyses of surgical outcomes according to the surgical methods.

**Table 2:** Anatomic and functional outcomes of epiretinal membrane surgery according to the surgical methods

			ERM only s ICG (*n* = 12)	ERM/ILM s ICG (*n* = 16)	ERM/ILM c ICG (*n* = 13)	p_12_	p_23_	p_13_	p_*_
CMT (μm)		Baseline	442.1 ± 64.2	444.4 ± 74.3	444.6 ± 95.4	0.931	0.994	0.938	0.996
		6 month	404.5 ± 51.0	389.2 ± 58.2	401.1 ± 47.3	0.466	0.549	0.864	0.719
		1 year	391.1 ± 42.0	373.5 ± 53.1	371.3 ± 55.6	0.337	0.915	0.325	0.571
BCVA (logMAR)		Baseline	0.43 ± 0.25	0.40 ± 0.19	0.44 ± 0.18	0.782	0.601	0.894	0.890
		6 month	0.15 ± 0.07	0.16 ± 0.10	0.19 ± 0.14	0.738	0.566	0.397	0.659
		1 year	0.13 ± 0.05	0.15 ± 0.09	0.15 ± 0.15	0.474	0.974	0.690	0.874
Microperimetry (dB)	MRS	Baseline	26.60 ± 1.11	25.89 ± 1.26	26.00 ± 0.91	0.125	0.789	0.154	0.228
		6 month	27.56 ± 1.23	26.45 ± 1.92	25.73 ± 1.37	0.075	0.249	0.002	0.022
		1 year	27.56 ± 0.72	26.83 ± 2.08	26.23 ± 1.01	0.204	0.322	0.001	0.092
	SN	Baseline	27.17 ± 1.70	25.88 ± 1.59	26.08 ± 1.55	0.052	0.733	0.109	0.102
		6 month	27.50 ± 1.09	26.75 ± 2.14	25.92 ± 1.55	0.239	0.240	0.007	0.083
		1 year	27.17 ± 1.53	26.88 ± 1.82	26.38 ± 1.71	0.649	0.462	0.239	0.513
	IN	Baseline	26.58 ± 1.00	26.25 ± 1.81	26.08 ± 1.32	0.540	0.768	0.288	0.682
		6 month	27.33 ± 1.78	26.63 ± 1.93	25.08 ± 2.47	0.324	0.077	0.015	0.028
		1 year	27.08 ± 0.51	27.13 ± 3.03	25.62 ± 1.26	0.958	0.085	0.001	0.109
	ST	Baseline	26.42 ± 1.98	25.81 ± 1.38	26.38 ± 1.26	0.376	0.254	0.962	0.499
		6 month	27.83 ± 1.70	26.56 ± 2.48	25.85 ± 1.82	0.120	0.378	0.010	0.065
		1 year	28.50 ± 1.57	27.00 ± 3.08	26.23 ± 1.01	0.106	0.359	0.000	0.041
	IT	Baseline	26.25 ± 1.96	25.63 ± 1.45	25.46 ± 1.20	0.364	0.743	0.245	0.414
		6 month	27.58 ± 1.83	25.88 ± 3.26	26.08 ± 2.53	0.091	0.853	0.101	0.223
		1 year	27.50 ± 1.45	26.31 ± 2.82	26.69 ± 2.14	0.161	0.683	0.278	0.397

Data are total no. (%) or mean ± standard deviation, unless otherwise indicated

*ERM* epiretinal membrane, *ILM* internal limiting membrane, *ICG* indocyanine green dye, *ERM only s ICG* ERM removal only without ICG staining group, *ERM/ILM s ICG* ERM/ILM removal without ICG staining group, *ERM/ILM c ICG* ERM/ILM removal with ICG staining group, *CMT* central macular thickness, *BCVA* best corrected visual acuity, *logMAR* logarithm of the minimum angle of resolution, *dB* decibel, *MRS* mean retinal sensitivity, *SN* superonasal area, *IN* inferonasal area, *ST* superotemporal area, *IT* inferotemporal area

## Discussion

In this study, CMT, retinal sensitivity, and visual acuity improved after combined ERM and cataract surgery, and retinal sensitivity was reduced when ILM removal was performed with, relative to without, the ICG dye.

PPV for ERM has been considered safe and effective [16]. Despite the surgical risks of PPV, such as retinal breaks and detachment, glaucoma, and endophthalmitis, PPV for ERM can have good anatomic and functional outcomes [16]. The progression of cataract after PPV is a well-known consequence of lens-sparing PPV, especially above the age of 50 years [17]. Therefore, combined ERM and cataract surgery has been widely performed recently. Some researchers are concerned that the rate of post-surgical macular edema is likely to be higher for combined ERM and cataract surgery than for lens-sparing PPV due to pro-inflammatory mediators from the anterior segment; however, evidence is still limited [16].

Baseline characteristics, including age, CMT, BCVA, and MRS, were not significantly different according to the surgical method used in this study (p > 0.05). Aging is a well-known factor for decreasing retinal sensitivity [18]. CMT is associated with ERM severity [19]. BCVA and retinal sensitivity are significant indicators of cataract severity and ERM. This means that the confounding factors were equally distributed among the groups in this study.

In this study, surgical outcomes, including CMT, BCVA, and retinal sensitivity, improved after surgery (*p* < 0.05). Yang et al. reported that CMT improved after ERM surgery [20]. Pesin et al. reported that BCVA improved after ERM surgery [21], and Vecchio et al. reported that retinal sensitivity improved after ERM surgery [3]. Some studies have reported decreased retinal sensitivity and microscotomas after ERM surgery with ILM peeling due to possible mechanical trauma from the forceps [4, 22]. However, in this study, decreased retinal sensitivity was not observed in all three groups. Intraocular pressure was stable pre- and postoperatively, and possible complications of PPV, including retinal breaks, detachment, and endophthalmitis, have not been reported.

ILM peeling has been widely used in ERM surgery [23, 24]. This procedure can lower the recurrence of ERM [23]. However, ILM removal can cause damage to the retina including the swelling of arcuate nerve fiber layer [25] and dissociated optic nerve fiber layer [26]. Therefore, ILM removal during ERM surgery is still controversial. The rate of recurrence of ERM after surgery is estimated to range from 1 to 16% [16]. In this study, ERM recurred in two patients, and both belonged to the “ERM removal only” group.

Indocyanine green has been used to visualize the ILM during vitrectomy [16]. However, ICG toxicity has been reported in in vitro and in vivo studies [27]. The mechanisms of ICG toxicity are unclear, but increased light absorption of the retina and stiffness of the membrane were observed [27]. In this study, ILM removal or the use of the ICG dye did not affect retinal sensitivity independently. However, ILM removal using ICG appeared to have a significant effect on retinal sensitivity. These results may be attributed to the toxicity of residual ICG after ILM peeling. Retinas with removed ILMs are prone to damage from the ICG dye. Uemura et al. reported peripheral visual field defects after ICG-assisted ILM peeling [28]. ICG demonstrated dose-dependent toxicity to the retinal ganglion cell [29]. Therefore, residual ICG after ILM peeling may show significant toxicity. Further studies are necessary to measure residual ICG in the retina.

This study is the first to evaluate ERM surgery using microperimetry to determine the combined effect of ILM removal and the ICG dye. Our study suggests that ILM removal with ICG staining can decrease retinal sensitivity without the deterioration of visual acuity.

This study had several limitations. First, it was retrospective. Therefore, we were unable to randomize the patient groups. To check the presence of selection bias, the baseline characteristics were compared between groups. However, baseline characteristics including CMT, BCVA, and microperimetry results were not significantly different between groups. Further prospective study is necessary to confirm. Second, the sample size was small. Therefore, the statistical power may not have been sufficient to observe this difference. Further studies with a larger sample size are required to confirm this hypothesis. Third, preoperative grading of cataract was not done, and its effect on visual acuity and retinal sensitivity might be a confounder in this study.

In conclusion, this study showed that retinal sensitivity was reduced when ILM removal was performed using the ICG dye.

## Data Availability

The datasets generated and/or analysed during the current study are not publicly available due to privacy or ethical restrictions but are available from the corresponding author on reasonable request.
